# Breast Imaging Chameleon: Pseudoangiomatous Stromal Hyperplasia Presenting as Breast Malignancy

**DOI:** 10.7759/cureus.8359

**Published:** 2020-05-30

**Authors:** Rabail Raza, Kulsoom Fatima, Muhammad Usman Tariq

**Affiliations:** 1 Radiology, Aga Khan University Hospital, Karachi, PAK; 2 Pathology, Aga Khan University Hospital, Karachi, PAK

**Keywords:** breast, ultrasound, malignancy, pseudoangiomatous stromal hyperplasia, palpable lump, benign

## Abstract

Pseudoangiomatous stromal hyperplasia (PASH) is a benign mesenchymal proliferative lesion of the breast, often an incidental finding on breast biopsy specimens and rarely presents as a palpable lump. The case being reported is interesting as a lactating female presented with gross left breast enlargement due to a huge firm mass with skin thickening and palpable left axillary lymph nodes. A provisional diagnosis of left breast malignancy was made and the patient extensively worked up with ultrasound, CT scan, bone scan and core biopsy. The histopathology, however, revealed PASH of the breast. There was no invasive or in situ malignancy. The patient was successfully managed conservatively.

## Introduction

Pseudoangiomatous stromal hyperplasia (PASH) of the breast, as the name suggests, is a benign process which on histology typically consists of anastomosing slit-like spaces within a background of dense fibrous stroma [1]. It is a usually a coexistent finding on histological examination of breast biopsy specimens and presentation as a palpable lump is rare. Since the first case reported in 1986 by Vuitch et al., approximately 150 cases of tumorous PASH have been reported in the literature [1,2]. The etiology of PASH is not clearly elucidated, but according to most investigators it represents a proliferative response of myofibroblasts under the influence of hormonal stimuli [3]. It, therefore, most commonly presents in premenopausal or perimenopausal women and may also be encountered in postmenopausal women receiving hormone replacement therapy [4]. Tumorous PASH presents as a slow-growing mass, with an average size of less than 7 cm and the clinical and radiologic features often suggest an alternate more common diagnoses, such as fibroadenoma, phyllodes tumor or hamartoma requiring histopathological confirmation [5,6]. As association with malignancy or malignant transformation is very rare, smaller incidental lesions may be periodically imaged to assess interval growth while surgical excision is reserved for larger symptomatic lesions [6].

## Case presentation

**Figure 1:**
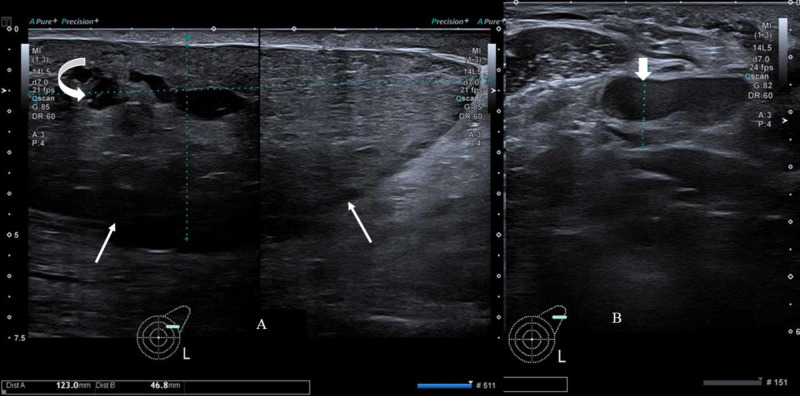
Gray scale ultrasound image of left breast and axilla (A) Image shows a solid, circumscribed mass (thin white arrows) predominantly in upper outer quadrant of left breast with few cystic spaces (curved arrow). (B) Image shows abnormal left axillary lymph node with asymmetric cortical thickening (short thick arrow) and displaced hilum.

A 30-year-old lactating female presented to the breast surgery clinic with a complaint of left breast heaviness and gradual enlargement since one year. Physical examination revealed gross asymmetric left breast enlargement with an ill-defined palpable lump measuring approximately 12 x 4.6 cm. There was overlying skin thickening with induration, although no redness and a palpable left axillary lymph node. As the patient was lactating, she initially underwent bilateral breast ultrasound, which showed a large circumscribed mass, wider than tall without any posterior acoustic shadowing predominantly in upper outer quadrant of the left breast with few intralesional cystic spaces showing mild vascularity on color Doppler and an abnormal left axillary lymph node (Figure 1).

The short axis of the lymph node measured 12 mm and the cortical thickness 8 mm with compressed fatty hilum. A breast imaging-reporting and data system (BI-RADS) category 4 assessment was given, and biopsy of the lesion and the axillary lymph node was recommended.

**Figure 2:**
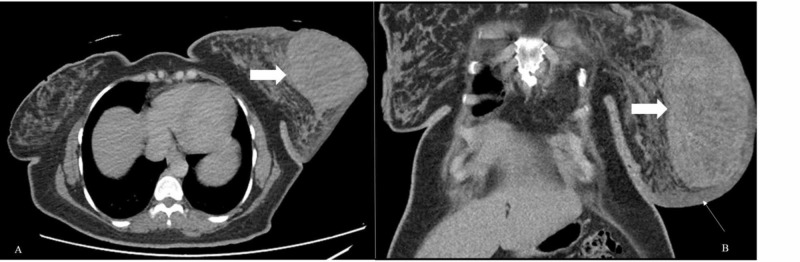
Contrast-enhanced CT scan Axial (A) and coronal (B) images show large enhancing soft tissue mass in the left breast (thick white arrows). Marked skin thickening is also noted (long white arrow) as well as left breast edema.

The patient was electively admitted for ultrasound-guided core biopsy, blood workup, CT chest, abdomen and pelvis scan, and bone scintigraphy for metastatic disease. Contrast-enhanced CT of the chest, abdomen and pelvis showed edematous left breast parenchyma with a large well-defined solid enhancing lesion associated with overlying skin thickening (Figure 2).

**Figure 3:**
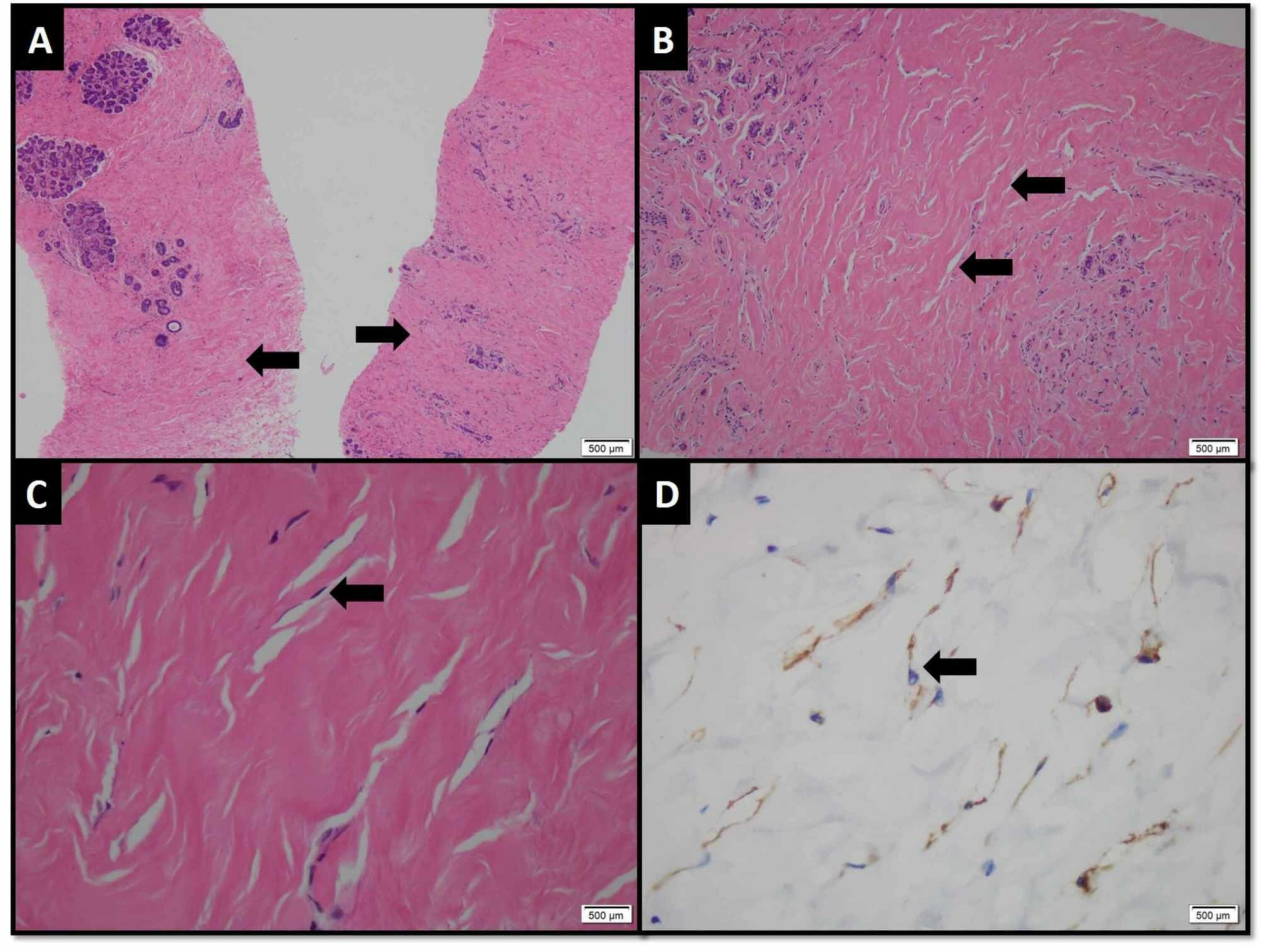
Photomicrograph (H&E and immunohistochemical stain) (A) Linear cores of benign breast tissue exhibiting ducts and lobule with surrounding hyalinized stroma (arrows. (B) Slit-like spaces mimicking thin blood vessels frequently seen in stroma (arrows). (C) These spaces are line by bland spindle shape cells (arrow). (D) The lining cells demonstrate positive expression for CD-34 immunohistochemical stain.

Multiple enlarged left axillary lymph nodes were also noted associated with perinodal fuzziness. There was no visceral metastasis on CT scan. Whole-body skeletal scintigraphy was also negative for bony metastasis. On histopathology, the core specimen from left breast lesion revealed benign breast tissue exhibiting adenosis, stromal sclerosis and pseudovascular proliferation of mammary stroma delineated by endothelial cells without atypia, features representing PASH (Figure 3).

There was no evidence of ductal carcinoma in situ (DCIS) or malignancy. Core specimen from the left axillary lymph node showed benign lymphoid tissue exhibiting sinus histiocytosis and acute on chronic inflammation.

The patient received lactation suppression drug (bromocriptine) and anti-hormonal therapy comprising tamoxifen, and she showed significant clinical improvement.

## Discussion

PASH is a benign entity characterized by a network of anastomosing slit-like spaces lined by spindle-shaped cells in a background of dense, collagenous proliferating breast stroma [2,3]. A proposed etiology is that this lesion is an abnormal response to progesterone and estrogen leading to myofibroblast proliferation; hence, it is more commonly seen in premenopausal or perimenopausal women and postmenopausal women on hormone replacement therapy [4,7]. PASH can be incidentally found in biopsy specimens performed for other breast lesions. They have very low mitotic index and rarely manifest as a symptomatic lesion, most cases being incidentally detected on imaging [8,9]. When symptomatic, the clinical presentation may range from a single firm, mobile, lump to multiple nodules in about two-third of cases [2,3,8-11]. The imaging appearances often simulate other common benign tumors, such as fibroadenoma or phyllodes. Because of considerable overlap of imaging features, the definitive diagnosis is established on histopathology [9].

On mammography, the most common appearance is a high-density, circumscribed, non-calcified mass. Suspicious features such as architectural distortion and spiculated margins are exceptionally rare [8,9,12] . On ultrasound, PASH is usually seen as a well-defined hypoechoic solid mass. Rarely, it may have a heterogeneous appearance with some cystic areas [13]. Our patient had well-circumscribed mass with smooth margins on ultrasound with few cleft-like anechoic cystic areas; however, large size, skin thickening and enlarged axillary lymph nodes were suspicious for malignancy and prompted extensive workup. Mammography was not performed as the patient was lactating.

On MRI, tumorous PASH may appear as a well-defined lesion showing gradual progressive enhancement on post-contrast sequences mimicking fibroadenoma [4]. Non-mass enhancement is rarely reported [13]. Such enhancement may simulate DCIS requiring biopsy to confirm this benign entity, especially in high-risk patients.

The definitive diagnosis is established on biopsy. The histological features of PASH may be confused with low-grade angiosarcoma; however, angiosarcoma shows anastomosing spaces filled with red blood cells invading the adjacent breast parenchyma and the absence of collageneous stroma, unlike PASH [6,11]. The hallmark of PASH is dense stromal proliferation with multiple slit-like spaces lined by bland spindle-shaped cells mimicking tiny blood vessels. Also, the proliferating myofibroblasts show positive expression of CD34 immunohistochemical stain in PASH [6]. This feature was also observed in our case. 

The treatment of PASH may be either surgical or non-surgical. Surgery is usually reserved for large symptomatic lesions with pain or enlargement [3,4,14]. As malignant transformation is extremely rare, serial imaging follow-up for few years can be considered for cases that are small, incidentally discovered or when it is asymptomatic [4,6,9]. Given the possible etiological role of hormones, anti-hormonal therapy can also be considered as an alternative non-invasive approach in the management of tumor-forming PASH [14]. Our patient did not opt for surgery. She was prescribed lactation suppression drug as she was lactating along with tamoxifen and pain medication, and the lesion remarkably regressed in size on clinical follow-up. The patient unfortunately did not have any post-treatment imaging.

## Conclusions

PASH is an uncommon histologic finding in breast biopsy specimens, often an incidental discovery. Uncommonly, it may present as a palpable breast mass and be indistinguishable from other benign breast masses. Rarely, it may attain a size or clinical appearance where it may be confused with malignancy. A core needle biopsy confirms the diagnosis. Large symptomatic masses may require excision. Some cases, which are hormone responsive, may benefit from conservative management.
